# Mechanosensing Piezo channels in gastrointestinal disorders

**DOI:** 10.1172/JCI171955

**Published:** 2023-10-02

**Authors:** Sandip M. Swain, Rodger A. Liddle

**Affiliations:** Department of Medicine, Duke University, Durham, North Carolina, USA. Veterans Affairs Durham Health Care System, Durham, North Carolina, USA.

## Abstract

All cells in the body are exposed to physical force in the form of tension, compression, gravity, shear stress, or pressure. Cells convert these mechanical cues into intracellular biochemical signals; this process is an inherent property of all cells and is essential for numerous cellular functions. A cell’s ability to respond to force largely depends on the array of mechanical ion channels expressed on the cell surface. Altered mechanosensing impairs conscious senses, such as touch and hearing, and unconscious senses, like blood pressure regulation and gastrointestinal (GI) activity. The GI tract’s ability to sense pressure changes and mechanical force is essential for regulating motility, but it also underlies pain originating in the GI tract. Recent identification of the mechanically activated ion channels Piezo1 and Piezo2 in the gut and the effects of abnormal ion channel regulation on cellular function indicate that these channels may play a pathogenic role in disease. Here, we discuss our current understanding of mechanically activated Piezo channels in the pathogenesis of pancreatic and GI diseases, including pancreatitis, diabetes mellitus, irritable bowel syndrome, GI tumors, and inflammatory bowel disease. We also describe how Piezo channels could be important targets for treating GI diseases.

## Introduction

Mechanotransduction is essential for sensing touch, hearing, and autonomic functions such as blood pressure regulation, gastrointestinal (GI) motility, cell differentiation and development, muscle stretch, and vascular tone (1, 2). In a two-step process, cells first sense mechanical stimuli through ion channels, transmembrane adhesion receptors, sarcomeric proteins, and cell surface receptors (3–7). These mechanoreceptors then convert mechanical forces into electrochemical signals that trigger appropriate cellular responses. However, defects in mechanotransduction are found in various genetic and acquired diseases, ranging from muscular dystrophy, cancer, and cardiomyopathies to GI disorders (1, 8, 9). Highly specialized electromechanical organs, like the heart and GI tract, generate tissue-specific mechanical forces sensed by specialized mechanosensitive cells (7, 10). The layers of the GI tract, including the mucosa, submucosa, and muscularis mucosae, have distinct mechanical properties that depend on specialized mechanoreceptor cell types. Coordination of these mechanical activities is essential for the peristaltic reflex, segmentation, and migratory motor activity that facilitate the digestion, absorption, and propulsion of food. In the GI epithelium, distinct sensory cells, like enterochromaffin cells, secrete serotonin (also known as 5-hydroxytryptamine [5-HT]) in response to epithelial mechanical forces, and thereby regulate intestinal fluid secretion (7). Neuronal cells in the GI tract, like intrinsic primary afferent neurons and extrinsic sensory neurons, sense physical forces and convert them into electrical signals (11). Nonsensory cells like interstitial cells of Cajal, smooth muscle cells, and immune cells respond to physical stimuli by altering the inherent functions specific to each cell type, be it motility, synthesis, or secretion (12, 13). Thus, responding to mechanical forces is an inherent and fundamental process for normal GI functions. However, disturbed mechanotransduction in the GI tract has been implicated in diverticulosis, intestinal pseudo-obstruction (14), chronic constipation (15), visceral hypersensitivity (16, 17), functional dyspepsia (18), irritable bowel syndrome, colon cancer (19), inflammatory bowel disease (20), and even systemic conditions, like obesity (21).

The initial step in mechanotransduction involves the activation of cell surface receptors or ion channels. Cells termed mechanoreceptors that transmit their mechanical cues to sensory neurons are deemed mechanosensitive. Several mechanosensitive ion channels, such as L-type voltage-gated calcium channels (e.g., Ca_v_1.2), voltage-gated sodium channels (e.g., Na_v_1.5), Ca^2+^-activated large-conductance potassium channels (e.g., BK_Ca_), and nonselective cation channels (e.g., TRPV2, TRPV4, TRPC4, TRPC6, TRPC7), regulate GI motor function and secretion and are associated with motility disorders (7, 10). Mechano-gated TREK1 channels expressed in the smooth muscle in the ileum and colon and TRAAK channels in enteric neurons modulate gut motility (10, 22). In contrast to the GI tract, the mechanosensing properties of liver cells have not been studied extensively, partly because of the lack of sensory innervation within the liver parenchyma. However, abnormal physical forces within the liver influence the phenotype of hepatic stellate cells, hepatocytes, portal fibroblasts, and endothelial cells and, in turn, contribute to hepatic fibrosis and cancer (23–25).

Although it has been underappreciated, the pancreas is a highly mechanosensitive organ that is susceptible to mechanical injury (26–28). Slight manipulations of the pancreas during abdominal surgery or abdominal trauma may induce pancreatitis, and animal models demonstrated that activation of mechanosensors on pancreatic acinar cells was the root cause (29–33). Pancreatic stellate cells also express mechanically activated ion channels that are coupled to cell activation and pancreatic fibrosis (26, 33, 34). Surprisingly, pancreatic β cells possess mechanically activated ion channels that appear to be coupled to insulin secretion (35, 36).

Insights into the mechanism of mechanosensing have been revealed by the recent discovery of a class of evolutionarily conserved mechano-gated ion channels known as Piezo (37). Piezo channels are a family of mechanically activated ion channels consisting of Piezo1 and Piezo2. Piezo1 is a large cation channel that is highly permeable to Ca^2+^ (2, 38, 39) and is activated by mechanical forces including static pressure, fluid shear stress, and stretch (2, 33, 34, 39, 40). It is expressed primarily in nonsensory tissues including the vascular and lymphatic systems, lung, bladder, skin, GI tract, and pancreas (26, 41, 42). Piezo2 is typically expressed in tissues that respond to physical touch, including sensory neurons. The molecular structure of Piezo channels has been determined by cryo–electron microscopy. The predicted size of approximately 2,500 amino acids is composed of a three-bladed propeller-shaped homotrimeric structure, 38-transmembrane regions, and a central pore. Piezo1 and Piezo2 share nearly 42% sequence homology (37, 43, 44). Both Piezo1 and Piezo2 are fast-activating and inactivating channels (37, 45–47). Endogenous activators of Piezo channels have yet to be identified, but several studies suggest that alteration of membrane lipids could modulate Piezo1 gating properties (48). It is possible that cholesterol could modulate Piezo1 and Piezo2 channel activities via cholesterol-binding stomatin-like protein 3 (STOML3), which physically interacts with the channels (49). STOML3 oligomerization reversibly reduces the sensitivity of Piezo currents in sensory neurons and tunes Piezo channel sensitivity at molecular-scale stimuli relevant to delicate touch. Moreover, Piezo1 gating properties depend on the cell’s intrinsic fatty acid composition (48, 50, 51). Matrix stiffness can regulate the sensitivity and degree of activation of mechanical ion channels including Piezo1 (52–54). Specifically, the collagen IV network of extracellular matrix (ECM) increases the sensitivity of Piezo1 to mechanical stimuli (53).

Several gain-of-function and loss-of-function mutations in Piezos have been linked with various hereditary human diseases, including hereditary xerocytosis, congenital lymphatic dysplasia, and an autosomal recessive syndrome of muscular atrophy with perinatal respiratory distress. Altered Piezo channel signaling has been associated with a variety of physiological and pathophysiological processes, including cell division, axon growth and regeneration, erythrocyte volume regulation, vascular hyperpermeability (55), chronic obstructive pulmonary disease, and pressure-induced pancreatic and renal (56, 57) fibrosis, touch, proprioception, pain, baroreceptor reflex regulation, urinary function, and innate immunity (34, 39, 47, 58–77). In addition, Piezo channels play an important role in tumor development and cancer cell metastasis (73, 78–81).

Piezo channels are expressed in many cells of the GI tract and are essential in proliferation, differentiation, secretion, and motility. This Review focuses on our current understanding of Piezos in GI function and disease. Studies using cell- or tissue-specific Piezo channel gene deletion in mice, siRNA-mediated gene silencing of Piezo channels, or pharmacological blocking of Piezo channels in vitro indicate that Piezo channels have a crucial role in GI cancer, fibrosis, inflammation, pain, secretion, and immunity.

## Mechanotransduction in pancreatic disease

Physical manipulation of the pancreas by surgery or endoscopic pancreatography may induce pancreatitis. The latter condition can be modeled experimentally by artificial increasing of pancreatic duct pressure (82). In the process of isolating pancreatic acinar cells, we noticed that mechanically disrupting the pancreas activated calcium-sensitive 5-lipoxygenase (26). Positing that this must be the result of increased intracellular calcium ([Ca^2+^]_i_), we discovered that Piezo1, an inherently mechanosensitive channel, is abundantly expressed in the pancreas (26, 37). Two approaches were used to assess the mechanosensitivity of pancreatic acinar cells. First, using a micropipette, mechanical force was applied to the surface of acinar cells loaded with a calcium-sensitive dye (33). Not only did touching a cell produce a rapid increase in [Ca^2+^]_i_, but the calcium signal was relayed to adjacent cells in a sequential manner consistent with the unit functionality of an acinus (33). Screening a library of 3 million compounds, Patapoutian’s group identified a selective Piezo1 agonist, Yoda1 (83). When applied to pancreatic acini, Yoda1 induced an increase in [Ca^2+^]_i_, reproducing the effects of mechanical force. Thus, Piezo1 confers mechanical sensitivity to pancreatic acinar cells through its calcium channel properties.

### Acute pancreatitis.

**Figure 1:**
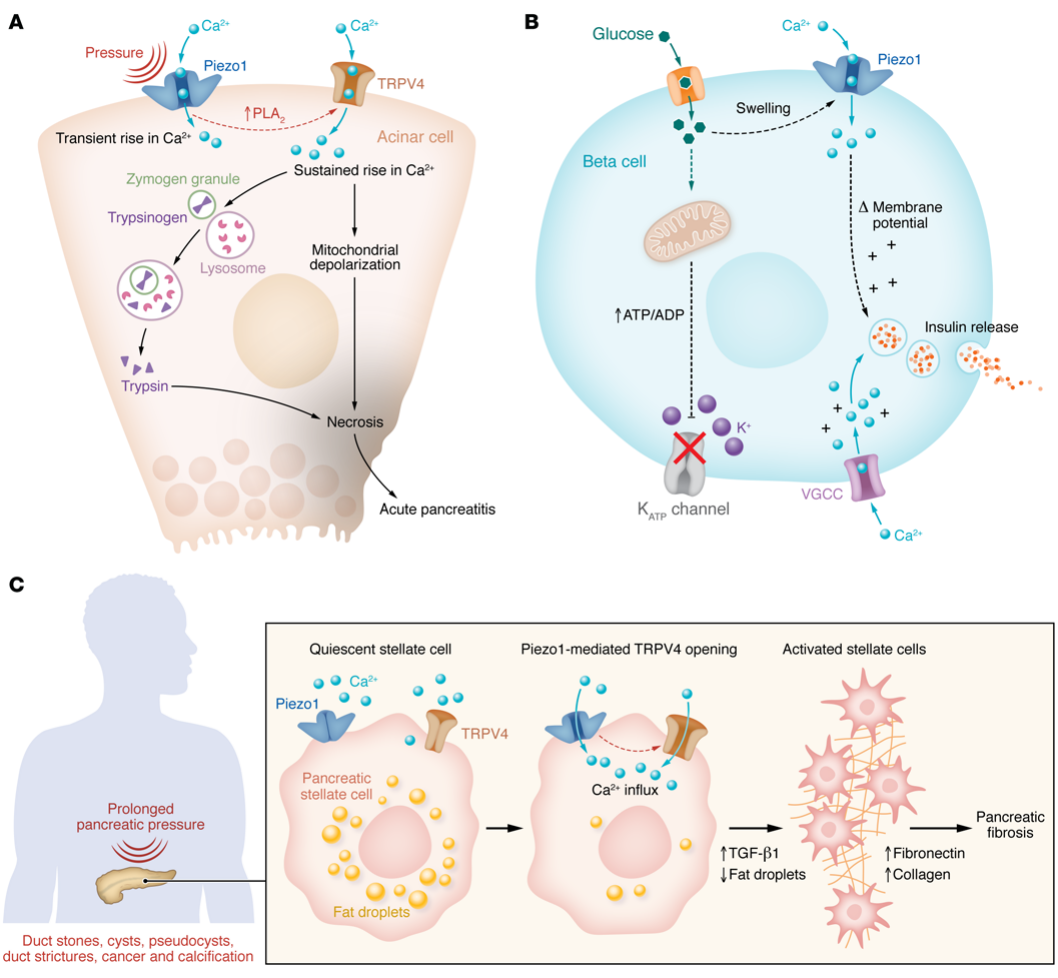
Model of mechanosensing in the pancreas. (**A**) Piezo1-induced TRPV4 opening causes intracellular calcium ([Ca^2+^]_i_) elevation in acinar cells and is responsible for pressure-induced pancreatitis. Pressure or shear stress force opens Piezo1 channels, which induces phospholipase A_2_ (PLA_2_) and activates TRPV4. Sustained elevation in [Ca^2+^]_i_ leads to mitochondrial depolarization, lysosome–zymogen granule fusion, trypsinogen activation, and pancreatitis. (**B**) Piezo1-induced [Ca^2+^]_i_ elevation through glucose-triggered dynamic changes in volume and deformation in cell membrane facilitates insulin release from pancreatic β cells. (**C**) Pressure-sensing Piezo1 channel signaling activates PSCs. The activated phenotype lacks perinuclear fat droplets and shows increased levels of TGF-β1, fibronectin, and type I collagen that contribute to pancreatic fibrosis. VGCC, voltage-gated calcium channel.

In pancreatic acinar cells, prolonged elevation in intracellular calcium concentration ([Ca^2+^]_i_) disrupts mitochondrial function, reduces ATP synthesis, and initiates zymogen granule–lysosome fusion and intracellular enzyme activation leading to pancreatitis (84, 85). Therefore, for self-preservation, acinar cells possess multiple safeguards to ensure [Ca^2+^]_i_ homeostasis. Once Piezo1 was discovered in pancreatic acini, it seemed possible that excess mechanical stimulation could cause pancreatitis through a calcium-sensitive mechanism (Figure 1), implying that precise regulation of [Ca^2+^]_i_ was necessary to prevent pancreatitis.

To evaluate this possibility, pressure-induced pancreatitis was produced in mice via pancreatic duct injection, a model of clinical pancreatitis that occurs as a complication following endoscopic retrograde cholangiopancreatography (ERCP) (26). Pancreatitis was blocked by treatment of mice with the Piezo1 inhibitor GsMTx4 (26, 86). Global deletion of *Piezo1* is embryonic lethal because of the wide distribution of Piezo1 in many tissues, including vascular endothelium, but conditional deletion of *Piezo1* in pancreatic acinar cells did not cause any detectable defect (26, 61). Importantly, mice with acinar cell–selective *Piezo1* knockout were protected against pressure-induced pancreatitis. The Piezo1 agonist Yoda1 increased [Ca^2+^]_i_ in pancreatic acinar cells and, in high concentrations, caused a sustained [Ca^2+^]_i_ elevation that led to sustained mitochondrial depolarization, trypsinogen activation, and lactate dehydrogenase (LDH) release, reproducing many features of pancreatitis in vitro (26, 33) (Figure 1A).

Since Piezo1 is a rapidly inactivating channel, Piezo1 opening produces only transient elevation in [Ca^2+^]_i_ that alone was insufficient to cause pancreatitis. Therefore, it was not known how pressure produced the prolonged [Ca^2+^]_i_ elevation necessary to induce pancreatitis. A series of in vitro and in vivo studies showed that the amount and duration of pressure applied to the pancreas determined the level and duration of [Ca^2+^]_i_ elevation in acinar cells and corresponded to pathological changes, e.g., intracellular trypsinogen activation and LDH release (33). Moreover, Piezo1 activation, caused by high pressure of long duration, triggered transient receptor potential vanilloid 4 (TRPV4) channel opening by activating phospholipase A_2_ (PLA_2_) and stimulating the conversion of arachidonic acid to epoxyeicosatrienoic acid, an endogenous activator of TRPV4 channels. In other words, the pathological consequences of pressure required TRPV4 activation (33, 87, 88) (Figure 1). Pressure-induced pancreatitis was reduced in mice with either acinar cell–specific deletion of *Piezo1* or global deletion of *Trpv4*. Thus, pressure-induced pancreatitis is caused by Piezo-initiated calcium signaling that requires TRPV4 to become fully manifest (33).

### Chronic pancreatitis and fibrosis.

Chronic pancreatitis is caused by persistent or repetitive injury to the pancreas and is characterized pathologically by gland atrophy, inflammatory cell infiltration, and fibrosis (89–91). Pancreatic fibrosis consists largely of collagen, fibronectin, and other ECM proteins that are produced and secreted by activated pancreatic stellate cells (PSCs) (92–95). Pancreatic fibrosis also develops under conditions of elevated pancreatic pressure, such as prolonged pancreatic duct obstruction or cancer, raising the possibility that pressure induces fibrosis and that PSCs are pressure sensitive (28, 34, 90, 96, 97). Consistent with this idea, PSCs were found to express Piezo1 (34). In a [Ca^2+^]_i_-dependent manner, shear stress or the Piezo1 agonist Yoda1 transformed PSCs from a quiescent to an activated phenotype characterized by loss of perinuclear fat droplets and increased TGF-β1, fibronectin, and type I collagen expression (34) (Figure 1). Like acinar cells, human and mouse PSCs also express TRPV4. Although TRPV4 does not have direct mechanosensing properties, in PSCs it is linked to Piezo1; and mice with either stellate cell–specific deletion of *Piezo1* or global *Trpv4* deletion were protected against pressure-induced pancreatic duct fibrosis (34). Thus, mechanical forces directly activate PSCs and induce pancreatic fibrosis.

### Piezo1-mediated endothelial dysfunction.

One-quarter of pancreatitis patients develop vascular complications, which contribute to the high mortality of severe pancreatitis (98, 99). Vascular hyperpermeability is a critical feature that promotes edema and vascular collapse, and severe pancreatitis can be complicated by splanchnic venous system thrombosis (100, 101). Piezo1 is highly expressed in vascular endothelium and may be activated by vascular shear stress or elevated intravascular pressure, leading to endothelial dysfunction (39, 102). We recently reported that Piezo1 mediates pathological endothelial cell responses to high-shear stress (39). Activation of Piezo1 by prolonged shear stress in endothelial cells produced a sustained elevation in [Ca^2+^]_i_ and increased PLA_2_ activity, which in turn caused TRPV4 channel opening, leading to cytoskeletal disorganization, increased expression of the adhesion protein VCAM1, and loss of adherens junctions. This disassembly of adherens junctions and elevated VCAM1 promoted leukocyte adhesion (39). These findings suggest a mechanism by which pathological Piezo1 activation within the endothelium may contribute to the vascular complications and edema that accompany acute pancreatitis.

### Piezo regulation of β cell function.

Glucose is the major physiological stimulant of insulin secretion from pancreatic β cells. Glucose and hypotonicity also induce cell swelling. Piezo1 was recently identified in β cells (35), and through a [Ca^2+^]_i_-dependent mechanism Piezo1 activation was shown to stimulate insulin secretion in β cell lines and isolated mouse islets (36) (Figure 1). Although the extent to which cell swelling contributes to insulin secretion in vivo is unknown, the observation that β cell–specific *Piezo1*-knockout mice exhibited impaired glucose tolerance suggests that Piezo1 may have a physiological role in insulin secretion. Interestingly, Piezo1 expression is elevated in islets from humans with type 2 diabetes and in the db/db mouse model of diabetes, in which Piezo1 translocates from the plasma membrane to the nucleus (36). Translocation of Piezo1 eliminates Piezo1-induced Ca^2+^ influx, and insulin secretion is reduced.

## Mechanosensing in the gut

GI motility is critical for the ingestion, propulsion, and digestion of food. The GI tract is composed of several types of mechanosensitive cells, including enterochromaffin cells of the mucosa, enteric neurons, smooth muscle cells (SMCs), and interstitial cells of Cajal (12). These cells express an array of voltage-gated calcium and sodium channels, potassium channels, and nonselective cationic channels that appear to respond to mechanical forces but lack inherent mechanosensing properties intrinsic to the channel itself. For this discussion, we refer you to several comprehensive recently published reviews on these channels (7, 10, 12, 13, 17, 41, 103–105).

Piezo channels are expressed in the epithelia, enteric nervous system, and SMCs of the digestive system as well as throughout the vascular smooth muscle and endothelium in humans and mice (10, 12, 13, 70, 106, 107). Gd^3+^, a nonspecific mechanical ion channel blocker, inhibits Piezo-type current in SMCs of the GI tract, suggesting a potential role of Piezo1 channels in peristalsis and other stretch-related functions in the gut (12, 108). Little is known about Piezo in myenteric plexus and intestinal muscularis cells (12). In the intestine, most epithelial cells, including goblet cells, Paneth cells, enterocytes, and endocrine cells, respond to mechanical forces, including stretch/distension, membrane distortion and deformation, shear stress, touch, tensile force compression, intraluminal pressure, and cell volume changes (7, 12, 109–111). Static forces in the gut are essential for epithelial cell proliferation, differentiation, and turnover (60). Piezo1 is present in most epithelial cells in the gut and can regulate epithelial cell proliferation and differentiation (112, 113). In the *Drosophila* gut, Piezo1 triggers the differentiation of stem cell progeny (112). In addition, Piezo1 controls normal epithelial homeostasis by inducing the extrusion of live cells that maintain the number of cells in the epithelia during proliferation-induced overcrowding (113). In contrast to Piezo1, Piezo2 is expressed in sensory neurons and primarily functions as a nociceptor (2, 46, 47, 114–118). However, it also regulates light touch (76) and is an important sensor in the GI tract.

### Piezo regulation of food intake.

External sensory cues and internal metabolic states control animal feeding. In *Drosophila*, Piezo-expressing neurons innervating the crop (equivalent to the mammalian stomach) sense mechanical cues of stomach fullness and prevent food overconsumption (119, 120). These neurons reside in the pars intercerebralis, a neurosecretory center in the brain, and express insulin-like peptides that regulate food intake and metabolism. *Piezo* knockdown led wild-type flies to eat more and exhibit an overconsumption phenotype similar to that of *piezo*-null mutant flies. Expression of either mammalian *Piezo1* or fly *piezo* in these neurons of *piezo*-null mutants prevented the overconsumption phenotype (119).

Nutrient sensors in the gut identify food rich in nutrients. A subset of *piezo*-expressing neurons also express diuretic hormone 44 (DH44), which helps flies detect sugar and operate during starvation. *Piezo* knockdown in DH44 neurons, which also project to the *Drosophila* crop, stimulates DH44 neuronal activity and food intake in fed flies (121). Together these observations indicate a role for Piezo channels in the regulation of satiety and food intake and open similar possibilities in mammals.

### Piezo2 in esophageal peristalsis.

Esophageal peristaltic movements are controlled by reflexes executed by vagal motor nuclei in the hindbrain, nucleus ambiguus, and esophageal enteric ganglia (122, 123). Clinically, esophageal dysmotility is typified by dysphagia, although the etiologies are diverse (122). The esophageal epithelium is innervated by several types of mechanosensory neurons with distinct electrophysiological properties and region-specific nerve endings such as intramuscular arrays, mucosal endings, and intraganglionic laminar endings (123). Single-cell RNA sequencing analysis identified two transcription factors, Prox2 and Runx3, that distinguish most vagal nodose neurons positive for Piezo2 (123). Three subtypes of Prox2 and Runx3 vagal neurons are low-threshold mechanoreceptors that innervate the esophagus and stomach, forming intraganglionic laminar endings on the enteric ganglia. Ablation of Prox2 and Runx3 neurons using intersectional genetic tools, followed by videofluoroscopic swallowing studies, in freely behaving rodents showed that esophageal motility requires these neurons (123). Esophageal intramuscular array neurons positive for Piezo2 also respond to stretch. It appears that Piezo2-positive vagal neurons respond to mechanical signals in the esophagus and control peristalsis, raising the possibility that they could have a role in dysphagia.

### Piezo in gut motility, secretion, and visceral sensitivity.

Enterochromaffin cells are a subtype of enteroendocrine cells and the primary source of serotonin. It has long been known that mechanical forces release serotonin, which stimulates GI motility and activates serotonin-sensitive colonic afferent neurons (124). However, it was not until the discovery of Piezo2 in enterochromaffin cells that it was appreciated that these effects were mediated through cell surface mechanically activated ion channels (70, 111). It was recently shown that applying a Piezo2 blocker or genetic deletion of *Piezo2* in enterochromaffin cells reduced stretch-induced Ca^2+^ signaling, serotonin release, and intestinal secretion (70, 111).

Irritable bowel syndrome (IBS) is a constellation of abdominal pain and alterations in bowel motility with diarrhea, constipation, or both. The underlying cause is unknown, although heightened visceral nociception is a prominent feature in many patients. Enterochromaffin cells communicate with enteric nerves through paracrine or synaptic connections comprising a semiautonomous effector network coupled to the parasympathetic and sympathetic nervous systems (125, 126). This bidirectional gut-brain connection involving serotonergic pathways influences both intestinal and extraintestinal symptoms in IBS. Elevated levels of serotonin have been reported in IBS, and 5-HT3 receptor antagonists and 5-HT4 receptor agonists have been used to treat either diarrhea- or constipation-predominant IBS, respectively, indicating that serotonin is involved in the pathogenesis of IBS (126–129).

Piezo2 in enterochromaffin cells might be linked to the pathophysiology of IBS. Stretching or elevated pressure within the GI tract activates Piezo2 in enterochromaffin cells, which release serotonin; therefore, serotonin-induced symptoms could arise from either excessive force producing hyperalgesia, or increased sensitivity to normal force (allodynia) (130). It remains to be determined whether conditions such as prolonged pressure in IBS can alter Piezo2 expression and influence downstream serotonin signaling. Both Piezo1 and Piezo2 are expressed in the enteric nervous system of mice, guinea pigs, and humans (106). However, Piezo1 is absent in sensory afferents. In contrast, Piezo2 is extremely rare in enteric neural somata but is enriched in dorsal root ganglion (DRG) neurons (which receive sensory and nociceptive input and transmit it to nerves ascending to the CNS), including small-diameter unmyelinated neurons, suggesting a possible role in mechanonociception (106). Growing evidence suggests that Piezo2 is critical for visceral hypersensitivity. In a rat model, neonatal colonic instillation of acetic acid induced visceral hypersensitivity, which was blocked by Piezo2 knockdown in DRG neurons, indicating that Piezo2 mediated the hypersensitivity response (131). Loss-of-function mutation in Piezo2 protects against pain sensitization. In contrast, activating Piezo2 in sensory neurons induces noxious stimuli (46, 47, 129).

**Figure 2:**
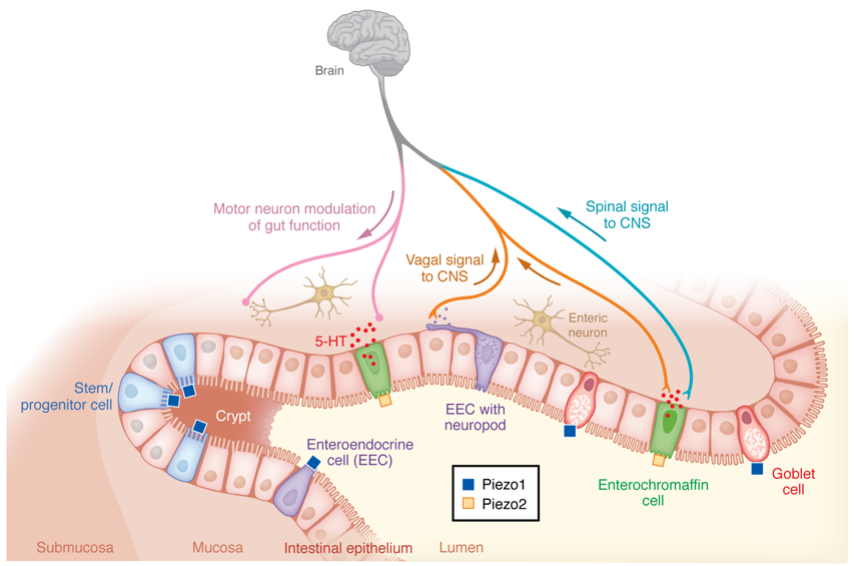
Schematic depiction of mechanosensing functions of Piezo1 and Piezo2 in the gut. Piezo1 is expressed on stem cells of the intestinal crypt, enteroendocrine cells (EECs), and goblet cells and stimulates stem cell proliferation, EEC differentiation, and mucus production, respectively. Piezo2 in enterochromaffin cells triggers 5-HT release and mediates visceral hypersensitivity.

In a classic study in IBS patients, heightened nociception was demonstrated in response to colonic balloon distention (132). Visceral pain is transmitted to the spinal cord via visceral afferent neurons, a subtype of which express the ion channel TRPV1. TRPV1 has been implicated in pain originating in many tissues, including the intestine, pancreas, and bladder (133). Mice with genetic deletion of TRPV1 exhibited reduced sensitivity to colorectal distension (134–136). Interestingly, Piezo2 is expressed in TRPV1-lineage nociceptors in the colon (114). Ablating either *Trpv1* or *Piezo2* in TRPV1-expressing colonic neurons reduced action potential firing by visceral afferents and visceromotor response in mouse models of zymosan-induced IBS and partial colon obstruction, raising the possibility that Piezo2 and TRPV1 could be targets for treating visceral pain caused by mechanical distension or stretch and may be relevant to visceral pain conditions such as inflammatory bowel disease, IBS, or intestinal obstruction (114) in which either excessive stretch or hypersensitivity to normal stretch may occur (Figure 2).

### Piezo1 in GI cancer.

Mechanical forces are essential for normal epithelial function. In contrast, abnormalities in force or altered sensation can lead to disease, including cancer (7, 19, 137). A unique characteristic of Piezo1 in the GI epithelium is its ability to sense cell crowding (113) and regulate cell migration (137). In GI epithelium, tumor growth exerts pressure on the adjacent epithelium. This altered mechanical stress induces aberrant crypt foci (19), suggesting that stem cell differentiation and cell migration are sensitive to surrounding forces (45, 68, 138).

Piezo1 channels are expressed in squamous cells of the esophageal mucosa, and recent studies demonstrated that Piezo1 levels are elevated in human esophageal squamous cell carcinoma (ESCC) tumors (139, 140). Mechanistically, Piezo1 promotes cancer cell metastasis and invasion by promoting cell migration, elevating the production of angiogenic factors, and enhancing matrix remodeling (54, 73, 78, 79, 81, 141, 142). These responses appear to be direct cellular effects of Piezo1, since downregulation of Piezo1 using shRNA inhibited the proliferation, migration, and invasion of ESCC cell lines EC109 and EC9706. Piezo1 downregulation inhibited epithelial-mesenchymal transition and restored the epithelial cell phenotype (139). Recent studies also indicate that Piezo1 plays a key role in cancer evolution. Downregulation of Piezo1 induced apoptosis via the Piezo1/p53/Bax/caspase-3 axis and inhibited G_0_/G_1_-to-S-phase cell cycle progression (139). In addition, Piezo1 monoclonal antibodies that induced Piezo1 internalization in human ESCC tumor cells when administered together with the antineoplastic agent monomethyl auristatin E preferentially killed ESCC tumor cells with high Piezo1 expression and suppressed tumor progression in ESCC xenograft tumor models, suggesting that Piezo1 may provide a novel target for ESCC (140).

Mechanical forces within tumors themselves regulate Piezo1 signaling that affects tumor growth and metastasis; and clinically, upregulation of Piezo1 expression in colon cancer is associated with a poor patient prognosis (143, 144). It appears that mechanical stress triggers Piezo1 signaling, leading to reduced expression of mitochondrial calcium uniporter, increased expression of HIF-1α and VEGF, and decreased mitochondrial membrane potential production, which promote colon cancer cell metastasis (143, 145, 146). Knockdown of Piezo1 channels by siRNA blocked the effects of mechanical stress to promote tumor malignancy and cancer metastasis in human colon cancer cell lines (HCT-116, SW-480) and similarly promoted gastric cancer in a xenograft model of human gastric tumors in BALB/c nude mice (143, 147).

### Immune cell actions of Piezo1.

Piezo1 has been linked to inflammation and immune surveillance in cancer (73, 80, 143, 147–150). Piezo1 is highly expressed in myeloid cells, where it is the primary mechanosensor linking mechanical stress to immune regulation. This function appears to be relevant to cancer surveillance, as deletion of Piezo1 in myeloid cells reduced pancreatic cancer progression (74, 151). Piezo1 is essential to the immune response in dendritic cells (151, 152). In a syngeneic ovalbumin mouse model, mechanical force upregulated production of the proinflammatory cytokines IL-6 and TNF-α in a Piezo1-dependent manner, and Piezo1-deficient dendritic cells suppressed the antitumor response (151).

In addition to dendritic cells, tumor-associated macrophages (TAMs) play a substantial role in cancer-related inflammation and are critical regulators of tumorigenesis, including gastric cancer (153, 154). Like many cancers, the gastric and colorectal tumor microenvironments harbor myeloid-derived suppressor cells, regulatory T cells, and TAMs and support tumor growth and progression (154–156). TAMs are derived from chemokine receptor type 2 inflammatory monocytes and consist of two main types, M1 and M2. M2 macrophages promote tumor progression by (a) triggering tumor neovascularization through the secretion of pro-angiogenic and inflammatory factors and (b) generating an immunosuppressive microenvironment. TAMs also can enhance tumor cell invasion and migration by promoting epithelial-mesenchymal transition and ECM remodeling (154). Furthermore, TAMs can reduce the effectiveness of cancer treatments, including chemotherapy, and response to immune checkpoint inhibitors and radiotherapy (154). Importantly, Piezo1 is necessary for macrophage polarization, raising the possibility that Piezo1 blockade might prevent the permissive effects of M2 TAMs (74, 150). The observation that genetic deletion of *Piezo1* in myeloid cells protected against cancer suggests that Piezo1 could be a target for suppressing inflammation in different organs, including GI tract cancers (74), although the potential for immunosuppressive side effects of such a strategy is unknown.

### Piezo1 in Crohn’s disease.

Crohn’s disease is a chronic intestinal inflammatory disease that can affect any region of the GI tract but most commonly involves the ileum and proximal colon. Initiation of proinflammatory responses in the intestine likely involves Piezo1, which is highly expressed in the ileum of patients with Crohn’s disease and positively correlates with the Crohn’s Disease Activity Index and fecal calprotectin levels (157). Activation of CD4^+^ T cells is a key feature in inflammatory bowel disease, and loss of Piezo1 in CD4^+^ T cells promotes Th1 and Th17 cell polarization. The subsequent reduction in inflammatory signals indicates that Piezo1 regulates the inflammatory response of pathogenic T cells. In a mouse model of chronic colitis, Piezo1 in CD4^+^ T cells was critical for development of intestinal inflammation (158), raising the possibility that Piezo1 activation could promote intestinal inflammation.

### Piezo1 in goblet cells.

Intestinal mucus is produced by goblet cells and limits the exposure of epithelial cells to bacteria. Defects in the gut mucus layer allow pathogenic bacteria to reach the epithelium and promote intestinal inflammation. Piezo1 is expressed in goblet cells and regulates mucus synthesis and secretion. Mice with goblet cell–specific Piezo1 knockout exhibited thinning of the intestinal mucus layer, elevated inflammatory cytokines (e.g., CXCL1, CXCL2, IL-6), and an increased number of pathogenic bacteria in the gut (159).

In humans, functional gut disorders are often linked with chronic stressful life events (160–162). Water avoidance stress has been used to model psychological stress in mice and induces colonic epithelial barrier dysfunction, colorectal hypersensitivity, and dysbiosis. It was recently shown in mice that water avoidance stress diminished Piezo1 expression in goblet cells, reduced mucus barrier function, and impaired intestinal motility (163). The Piezo1 agonist Yoda1 improved mucus barrier function and reversed the defect in intestinal motility. Although these studies point to a beneficial effect of Piezo1 on intestinal barrier function by maintaining the mucus layer, there is evidence that Piezo1 may also negatively regulate epithelial tight junction function (164). Overexpression of Piezo1 or stimulation of Piezo1 by Yoda1 decreased the tight junction protein claudin-1, leading to impaired colon epithelial integrity and loss of barrier function.

Overall, it appears that excessive Piezo1 activity has deleterious effects on intestinal immunity and epithelial tight junctions. Whether its positive effects maintaining intestinal mucus production outweigh these other actions remains to be determined.

## Conclusions

Mechanically activated ion channels detect physiological pressure, shear force, stretch, and stiffness, and promote normal biological responses including cell growth, migration, turnover, and secretion. Abnormally high or prolonged mechanical forces induce pathological changes that are manifested by cellular and tissue damage that initiates an inflammatory response. In the pancreas, acute pancreatitis occurs when acinar cells are subjected to excessive force, and fibrosis follows chronic stellate cell stimulation. The observation that Piezo1 is required for normal insulin secretion raises the intriguing possibility that mechanosensing may be a common feature of exocytosis in other cell types. Piezo channels in enterochromaffin cells link pressure and distension in the gut to the release of serotonin and its pathological consequences in IBS. By virtue of its expression in immune cells, Piezo1 contributes to intestinal inflammation and immune surveillance. Piezo channels are upregulated in GI cancers including gastric cancer and colorectal carcinoma, and, by virtue of their ability to sense cell crowding, these receptors may be involved in cancer metastasis. Despite the attraction of mechanoreceptor blockade as a possibility for treating or preventing inflammation or cancer, the wide distribution of Piezo channels in non-target tissues may limit the utility of Piezo-targeted pharmacotherapies. Perhaps development of tissue-selective therapies or therapies directed at pathways downstream of Piezo signaling would mitigate potential off-target effects.

## Version 1 10/02/2023

Electronic publication
